# Current status of immunotherapy against gastrointestinal cancers and its biomarkers: Perspective for precision immunotherapy

**DOI:** 10.1002/ags3.12180

**Published:** 2018-06-22

**Authors:** Shoichi Hazama, Koji Tamada, Yoshiyuki Yamaguchi, Yutaka Kawakami, Hiroaki Nagano

**Affiliations:** ^1^ Department of Translational Research and Developmental Therapeutics against Cancer Yamaguchi University School of Medicine Ube Japan; ^2^ Department of Immunology Yamaguchi University Graduate School of Medicine Ube Japan; ^3^ Department of Clinical Oncology Kawasaki Medical School Kurashiki Japan; ^4^ Division of Cellular Signaling Institute for Advanced Medical Research Keio University School of Medicine Tokyo Japan; ^5^ Department of Gastroenterological, Breast and Endocrine Surgery Yamaguchi University Graduate School of Medicine Ube Japan

**Keywords:** gastrointestinal cancer, immune checkpoint inhibitor, immunity, microenvironment, precision immunotherapy

## Abstract

Immunotherapy has shown encouraging results for some types of tumor. Although enormous efforts have been made toward the development of specific immunotherapeutic strategies against gastrointestinal cancers, such as adoptive T‐cell transfer, peptide vaccines, or dendritic cell vaccines, the efficacy of immunotherapies prior to the introduction of immune checkpoint inhibitors was not substantial. This article reviews immunotherapy for gastrointestinal malignancies, including cell therapy, peptide vaccine, and immune checkpoint inhibitors, and attempts to resolve the immunosuppressive conditions surrounding the tumor microenvironment, and to construct novel combination immunotherapies beyond immune checkpoint inhibitors.

## 1 INTRODUCTION

Gastrointestinal (GI) cancers are the most common human tumor worldwide, and the incidence and mortality are increasing every year.^1^ Several treatment strategies have been developed for GI cancers, including surgery, chemotherapy, radiotherapy, and molecularly targeted therapy. However, the overall survival (OS) of patients with GI cancer remains poor. Novel approaches to the treatment of GI cancer are thus needed.^2^

Immunotherapy is a novel treatment strategy that is emerging as an effective and promising treatment option against several types of cancer.^3^ The first immunological treatments were carried out by Coley using a bacterial immunotoxin to patients with malignancy in 1891.^4^ The first notion of a role of immunity in cancer was postulated in 1909 by Smith,^5^ speculating that the immune system could repress the growth of carcinomas by recognizing tumor cells as foreign. In 1970, the concept of immunological surveillance was presented by Burnet,^6^ and an antigen recognized by cytolytic T lymphocytes on a human melanoma was finally reported by van der Bruggen et al.^7^

Progress in this field is largely attributable to the identification of new immune‐based targets, based on continued advances in the understanding of tumor immunology and the tumor microenvironment.^8^ Many types of immunomodulatory therapies have been demonstrated in the treatment of GI cancers, including non‐specific biological response modifiers (OK432,^9^ lentinan,^10^ PSK^11^), interleukin (IL)‐2‐activated lymphocytes,^12^ tumor‐specific reactive CD8^+^ T‐lymphocyte transfer,^13^ dendritic cell (DC) vaccines,^14^, ^15^ and tumor‐associated antigen (TAA)‐derived peptides.^16^, ^17^, ^18^, ^19^ These immunotherapies have shown a certain degree of efficacy, but not durable objective responses.^20^ Confidence in the efficacy of immunotherapies was given a boost with the advent of immune checkpoint inhibitors, which was selected as a “Breakthrough of the Year 2013” by *Science*.^21^ Immunotherapy is now becoming mainstream as a treatment for GI cancer.

In the present study, we review immunotherapies for GI malignancies, including immune cell transfer therapy, peptide vaccine, immune checkpoint inhibitors, and combination immunotherapy beyond immune checkpoint inhibitors, by clarifying suppressive immune biomarkers surrounding the tumor microenvironment.

## 2 IMMUNOTHERAPY AGAINST GI CANCERS

### 2.1 Adoptive T‐cell transfer

The concept of adoptive immunotherapy (AIT) for cancer treatment was presented by Mule et al^22^ in the form of IL‐2 generated lymphokine‐activated killer (LAK) cells combined with repeated injections of recombinant IL‐2 (Table 1). Although LAK cells are non‐specific killer cells that were considered effective against various types of tumor, the efficacy of LAK cells combined with high‐dose IL‐2 proved limited against metastatic GI cancer. The objective response rate (ORR) including complete response (CR) or partial response (PR) for colorectal cancer (CRC) was 11% (3 of 27 patients), and 0% (0/1) for esophageal cancer. Furthermore, severe toxicities were observed as a result of high‐dose IL‐2, which induces a vascular permeability leak that leads to fluid retention and interstitial edema, and results in circulatory failure, lung edema, and renal dysfunction. Hence, they made the shift to tumor‐infiltrating lymphocytes (TIL), which are specific to tumor antigens and appear to offer far greater therapeutic potency than LAK cells.^28^

**Table 1:** Previous studies on gastrointestinal cancers discussed in the present review

Tumor type	Target	Key drug and study design	Treatment line	Phase	Allocation	Sample size	Clinical efficacy	irAE	Reference
CRC	Non‐specific	LAK with IL‐2	Late	Pilot	Review in an institute	27	3 PR of 27 (11%)	SAE as a result of high‐dose IL‐2: fluid retention, circulatory failure, lung edema, and renal dysfunction	22
HCC	Non‐specific	Activated killer cells	Adjuvant	II	Randomized	150	Longer DFS (*P* = .01) and DSS (*P* = .04) than control	No severe irAE	23
Liver tumor^a^	Autologous tumor	CTL, HAI	Late	Pilot	Retrospective	15	2 CR and 3 PR of 15 (33%)	No severe irAE	24
PDAC	MUC1	MUC1‐CTL and gemcitabine	Adjuvant	Pilot	Retrospective	21	DFS, 15.8 M, OS 24.7 M (median)	No severe irAE	25
PDAC	MUC1	MUC1‐CTL, MUC1‐DC, and gemcitabine	1st	Pilot	Retrospective	42	MST, 13.9 M; 1 CR (2.4%), 3 PR (7.1%) and 22 SD (52.4%)	No severe irAE	15
HCC	HSP70	HSP70‐DC	Late	I	Dose escalation	12	2 CR (17%), 5 SD	Grade 3 liver abscess	14
CRC	Oncoantigens	Peptide cocktail with IFA	Late	I	Dose escalation	18	MST, 13.5 M; 1 CR (6%), 6 SD (33%)	No severe irAE	26
CRC	Oncoantigens	FOLFOX + peptide cocktail with IFA	1st	II	One arm, HLA‐blind	96	ORR and OS did not differ from control group	Grade 5 IP, 2 in study group, 1 in control group	17
PDAC	Oncoantigens	Gemcitabine + peptide cocktail with IFA	Adjuvant	II	One arm	30	Median DFS, 15.8 M	No severe irAE	18
HCC	GPC3	GPC3 peptide with IFA	Adjuvant	II	One arm	41	Recurrence rate, 28.6% (1 year), 39.4% (2 years)	No severe irAE	27
CRC	Tumor specific	TSA with various adjuvants	Late	I/II	Review	527	1 CR and 4 PR (ORR, 0.9%)	Not evaluated	20

Liver tumor^a^: HCC 13 patients, CRC 2 patients.

CR, complete response; CRC, colorectal cancer; CTL, cytotoxic T cell; DFS, disease‐free survival; DSS, disease‐specific survival; GPC3, glypican‐3; HAI, hepatic arterial infusion; HCC, hepatocellular carcinoma; HLA, human leukocyte antigen; HSP, heat‐shock protein; HSP70‐DC, dendritic cells transfected with HSP70 mRNA; IFA, incomplete Freund's adjuvant; IL‐2, interleukin‐2; IP, interstitial pneumonia; irAE, immune‐related adverse effects; LAK, lymphokine‐activated killer cells; M, month; MST, median survival time; MUC1‐DC, dendritic cells transfected with MUC1 mRNA; ORR, objective response rate; OS, overall survival; PDAC, pancreatic ductal adenocarcinoma; PR, partial response; SAE, severe adverse event; SD, stable disease; TSA, tumor‐specific antigen.

Takayama et al conducted a randomized study to evaluate the efficacy of autologous lymphocytes activated in vitro with recombinant IL‐2 and solid‐phase antibody to CD3 as adjuvant therapy for curatively resected HCC. A total of 150 patients who had undergone curative resection for HCC were assigned to receive either AIT (n = 76) or no adjuvant treatment (n = 74). The immunotherapy group showed significantly longer DFS (*P* = .01) and disease‐specific survival (*P* = .04) than the control group. No patients experienced grade 3 or 4 adverse events.^23^ These results suggested that transfer of non‐specific‐activated killer cells might be effective in preventing the intrahepatic recurrence of cancer.

The next advance was antigen‐specific cytotoxic T lymphocytes (CTL) for the management of effector cells as treatment.^24^

In Japan, Aruga et al reported autologous tumor‐specific CTL, induced from peripheral blood mononuclear cells (PBMC) cultured with autologous tumor cells. These CTL were injected through the hepatic artery into patients with unresectable liver tumors. Among 15 treated patients (13 with hepatocellular carcinoma [HCC] and 2 with metastatic liver cancer), two CR, three PR, and four minor responses were observed without any severe treatment‐associated systemic adverse events.^29^

We have assessed the efficacy of CTL against pancreatic ductal adenocarcinoma (PDAC). Patients with curatively resected PDAC received AIT with CTL stimulated using MUC1‐expressing human cell lines (MUC1‐CTL), and the results indicated that MUC1‐CTL might prevent liver metastasis.^30^ For the next step, combination therapy using MUC1‐CTL and gemcitabine was carried out. A total of 43 patients who underwent radical pancreatectomy received treatment with MUC1‐CTL and gemcitabine after surgery. MUC1‐CTL were induced and given i.v. three times, and gemcitabine was given according to the standard regimen for 6 months. No severe treatment‐associated systemic adverse events were encountered in the 43 treated patients. In the adequate treatment group (n = 21) in which the relative dose intensity of gemcitabine was ≥50% and ≥2 MUC1‐CTL treatments were provided, disease‐free survival (DFS) was 15.8 months, and OS was 24.7 months. Liver metastasis was found in seven patients only (33%), and local recurrence occurred in four patients (19%). Combination therapy with AIT and GEM might prevent liver metastasis and local recurrence.^25^

As described above, adoptive immunotherapies have shown a certain degree of efficacy (Table 1). To obtain more effective arms, revolutions in technologies are needed; these include expanding neoantigen recognized TIL or genetically engineered T cells such as T‐cell receptor (TCR) T cells and chimeric antigen receptor (CAR) T cells.^31^

So‐called CAR‐T therapy was also selected as a “Breakthrough of the Year 2013” by *Science*.^21^ CAR‐modified T cells (CAR‐T) targeting CD19 showed durable effects against leukemia, achieving complete remission.^32^ For gastrointestinal tumor, CAR‐T therapies remain experimental.^33^ In clinical studies of CRC^34^ and biliary tract and pancreatic cancers,^35^ some promising results have been reported in the form of PR and long‐term stable disease without uncontrollable toxicities. The novel next‐generation CAR‐T therapy was reported by Adachi et al.^36^ They engineered CAR‐T cells to express IL‐7 and chemokine (C‐C motif) ligand 19 (CCL19) (7 × 19 CAR‐T cells), as these factors are essential for the maintenance of T‐cell zones in lymphoid organs. In mice, 7 × 19 CAR‐T cells achieved complete regression of pre‐established solid tumors with anti‐tumor activity superior to that of conventional CAR‐T cells. Histopathological analyses showed increased infiltration of DC and T cells into tumor tissues following 7 × 19 CAR‐T‐cell therapy,^36^ which might be adapted against gastrointestinal cancers in the near future.

### 2.2 Dendritic cell vaccines

Dendritic cells are antigen‐presenting cells specialized for the induction of a primary T‐cell response. A clinical pilot study reported generation of DC in the presence of granulocyte‐macrophage colony‐stimulating factor and IL‐4. These cells were pulsed with tumor lysate or a cocktail of TAA‐derived peptides. This method to induce DC has been used as a standard worldwide.^37^ The study analyzed 16 patients with advanced melanoma, and objective responses were evident in five of these 16 evaluated patients.

For patients with PDAC, the clinical efficacy of immunotherapy using both DC transfected with MUC1 mRNA (MUC1‐DC) and MUC1‐CTL was evaluated in a pilot study with gemcitabine. Forty‐two patients with unresectable or recurrent PDAC were enrolled, and median survival time was 13.9 months, with a 1‐year survival rate of 51.1%. Of the 42 patients, one patient achieved CR (2.4%), three patients had PR (7.1%), and 22 patients had stable disease (SD) (52.4%). The disease control rate (DCR) was thus 61.9%. No severe toxicities were associated with cell transfer. MUC1‐DC and MUC1‐CTL plus gemcitabine might offer an effective treatment for PDAC.^15^

Another target of DC therapy is HCC. A phase I trial was conducted on the basis of a previous basic study that reported overexpression of heat‐shock protein (HSP) 70 in HCC using proteomic profiling and immunohistochemical staining.^38^ DC transfected with HSP70 mRNA (HSP70‐DC) by electroporation were injected intradermally. Patients were treated three times every 3 weeks, and the number of HSP70‐DC injected was dose‐escalated in a three‐patient method, from 1 × 10^7^ to 2 × 10^7^, and finally to 3 × 10^7^. No adverse effects at grade III/IV were observed, except for one case of grade III liver abscess at the 3 × 10^7^ dose, and three patients were therefore added to confirm the safety of the 3 × 10^7^ dosage. CR without any recurrence was achieved in two patients (for at least 44 and 33 months) and SD in five patients. That study indicated that HSP70‐DC therapy is both safe and effective in patients with HCC.^14^

DC vaccines might gain a place in novel combination immunotherapy.

### 2.3 Peptide vaccines

Since the first clinical trial of a melanoma antigen gene‐1‐derived peptide‐based vaccine was reported in 1995,^39^ various types of next‐generation peptide vaccine are currently under development.^16^ Here, we present some successful reports from among these numerous studies.

We conducted phase I and phase II trials using HLA‐A*24:02‐restricted peptides, three derived from oncoantigens and two from vascular endothelial growth factor receptors (VEGFR) against CRC. In the phase I study, 18 HLA‐A*2402‐positive CRC patients for whom standard therapy had failed were enrolled, and 0.5 mg, 1.0 mg, or 3.0 mg each of the peptides was mixed with incomplete Freund's adjuvant (IFA) and then s.c. injected. Vaccine treatment was well tolerated without any severe treatment‐associated systemic adverse events.^26^ One patient who achieved CR remains alive without recurrence more than 10 years after the initial vaccinations, and six patients showed stable disease for 4‐7 months. Median overall survival time (MST) was 13.5 months.

The phase II study was conducted to evaluate the efficacy of this approach in combination with oxaliplatin‐based chemotherapy as a first‐line therapy. Ninety‐six chemotherapy‐naïve CRC patients with measurable metastatic or unresectable lesions were enrolled under masking of HLA‐A status. Although ORR and OS did not differ between the HLA‐A*2402‐matched and unmatched groups, a significantly delayed response was observed in the subgroup with a neutrophil‐to‐lymphocyte ratio (NLR) <3.0 according to the Harrington‐Fleming method. Although the incidences of serious adverse events (SAE) were broadly similar between groups, that of neutropenia was relatively higher in the HLA‐A*2402‐matched group than in the unmatched group. Interstitial pneumonia that led to death was observed in two cases in the HLA‐matched group and in one case in the HLA‐unmatched group.^17^

In the adjuvant setting, Miyazawa et al reported that 30 patients with resected PDAC were treated using a peptide cocktail vaccine containing epitope peptides derived from KIF20A, VEGFR1, and VEGFR2 combined with gemcitabine as a single‐arm multicenter phase II study. No serious (more than grade 3) immune‐related adverse events (irAE) were encountered. Median DFS was 15.8 months. This study also conducted comparisons with 15 patients treated using gemcitabine alone as a prospective control group that did not meet eligibility criteria as a result of HLA‐A type only, for whom the median DFS was 12.0 months. No significant difference was seen between the two groups (*P* = .504). Significant differences in DFS were apparent between patients with and without KIF20A‐specific CTL responses (*P* = .027), and between patients with and without KIF20A expression (*P* = .014). In addition, all four patients who underwent R0 resection with KIF20A expression showed no recurrence with KIF20A‐specific CTL responses.^18^

Sawada et al identified in glypican‐3 (GPC‐3) an HLA‐A*24, HLA‐A*02 restriction peptide with extreme cancer specificity. In a phase I study, they reported safety, and immunological and clinical responses.^40^ A subsequent trial showed a durable effect against giant HCC, although the patients died from circulatory failure as a result of tumor thrombus, which occupied most of the right atrium.^41^ In the next phase II study of GPC3 peptide vaccine as an adjuvant therapy for HCC, no significant difference in recurrence rate was found between 35 patients treated with surgery plus vaccination and 33 patients who underwent surgery alone (28.6% vs 54.3% at 1 year and 39.4% vs 54.5% at 2 years, respectively; *P* = .346, .983). In a subgroup analysis, 25 patients treated with vaccination showed GPC3‐positive tumors and a significantly lower recurrence rate compared to that in the 21 GPC3‐positive patients who received surgery alone (24% vs 48% at 1 year and 52.4% vs 61.9% at 2 years, respectively; *P* = .047, .387). GPC3 peptide vaccine improved the 1‐year recurrence rate in patients with GPC3‐positive tumors.^27^

Although tumor‐associated antigen‐derived peptide vaccines have been shown to provide effective induction of antigen‐specific immunity, the clinical efficacy has not proven durable (Table 1). As a result, no peptides are covered by the National Health Insurance. Pooled results of clinical trials show a very weak clinical response rate of <1% for the active specific immunization procedures currently available for advanced CRC.^20^ Rosenberg et al reported that the objective response rate was low (2.6%) in their cancer vaccine trials of 440 patients, even though the main target was melanoma, which is highly immunogenic.^42^

Combination immunotherapy appears needed for peptide vaccinations such as immune checkpoint inhibitors,^43^ novel immune adjuvants,^44^ COX‐2 inhibitors,^45^ and anti‐epidermal growth factor receptor (EGFR) antibodies.^46^ Such novel approaches are described in detail in later sections.

Neoantigens, a class of HLA‐bound peptides that arise from tumor‐specific mutations, are highly immunogenic because they are not present in normal tissues and hence bypass central thymic tolerance. Ott et al demonstrated the feasibility, safety, and immunogenicity of a vaccine targeting up to 20 predicted personal tumor neoantigens using machine learning approaches to reliably predict those mutated peptides with high‐affinity binding of autologous HLA molecules. Of six vaccinated patients, four showed no recurrence at 25 months after vaccination, whereas two with recurrent disease were subsequently treated with anti‐programmed cell death protein 1 (PD‐1) therapy and achieved CR, with expansion of the repertoire of neoantigen‐specific T cells. These data provide a strong rationale for further development of this approach alone and in combination with checkpoint blockade or other immunotherapies.^47^ Simultaneously, the first in‐human application of this concept was carried out in melanoma using an RNA‐based poly‐neo‐epitope approach comprising computational prediction of neo‐epitopes, and design and manufacturing of a vaccine unique to each patient. All patients developed T‐cell responses against multiple vaccine neo‐epitopes at up to high single‐digit percentages. Cumulative rate of metastatic events was significantly reduced after the start of vaccination, resulting in sustained progression‐free survival (PFS). Two of the five patients with metastatic disease experienced vaccine‐related objective responses. A third patient developed CR to vaccination in combination with PD‐1 blockade therapy.^48^ Although these reports involved melanoma, this strategy holds promise for the treatment of gastrointestinal cancers.

## 3 IMMUNE CHECKPOINT INHIBITORS

Recently, the effectiveness of immunotherapy targeting immune checkpoints in the treatment of numerous forms of cancer has been studied. In 2011, the Food and Drug Administration in the USA approved ipilimumab, an anti‐cytotoxic T‐lymphocyte‐associated antigen 4 (CTLA‐4) treatment for metastatic melanoma. In 2012, Topalian et al^49^ reported results for PD‐1 therapy in nearly 300 people, and an update was provided in 2013.^50^ Tumors shrunk by about half or more in 31% of those with melanoma, in 29% with kidney cancer, and in 17% with lung cancer. Immune checkpoint inhibitors have also been applied to gastrointestinal cancers (Tables 2, 3, 4).

**Table 2:** Published studies of immune checkpoint inhibitors for gastrointestinal cancers discussed in the present review

Tumor type	Target	Key drug and trial identifier	Treatment line	Phase	Allocation	Sample size	Clinical efficacy	irAE	Reference
ESCC	PD‐1	Nivolumab, ONO‐4538‐07	Late	II	Single arm	64	ORR, 11 of 64 (17%)	Lung infection (4), dehydration (2), IP (2) of 65No treatment‐related deaths	51
GC or GEJC	PD‐1	Nivolumab, ATTRACTION‐2	3rd or more	III	Randomized, double‐blind	493 (330 vs 163)	ORR, 30 (11.2%) of 268, 1Y OS: 26.2% (Nivo) vs 10.9% (Place)	Grade 3 or 4 irAE in 34 (10%); irAE led to death in 5 (2%)	52
PD‐L1 + GC or GEJC	PD‐1	Pembrolizumab, KEYNOTE‐012	Late	Ib	Single arm	39	ORR, 22% (8 of 36)	Grade 3, 2 fatigue, 1 PG, 1 hypothyroidism, 1 PSN; Grade 4, 1 IP No treatment‐related deaths	53
GC or GEJC	PD‐1	Pembrolizumab, KEYNOTE‐059‐Cohort 1	3rd or more	II	Single arm	259	ORR, 15.5% in PD‐L1+ pts, 5.5% in PD‐L1– pts	Grade 3‐5 irAE in 43 (16.6%), discontinuation in 2 (LFT, BDS), fatal in 2 (AKI, PE)	54
dMMR/MSI‐H CRC	PD‐1	Pembrolizumab, CT01876511	Late	II	Single arm	28	ORR, 40% (4 of 10) for dMMR/MSI‐H CRC, 0% (0 of 18) for pMMR CRC	Grade 3 and 4 irAE, anemia (17%), lymphopenia (20%), diarrhea (5%), BO (7%)	55
dMMR/MSI‐H tumors^a^	PD‐1	Pembrolizumab, CT01876511	2nd or more	II	Single arm	86	ORR, 53%; CR, 21%	irAE were manageable74% had AE (Grade 1 or more), hypothyroidism (21%) managed with THR	56
dMMR/MSI‐H CRC	PD‐1	Nivolumab, CheckMate 142	2nd or more	II	Single arm	74	ORR, 31.1% (23 of 74)	Common grade 3 or 4 irAE, elevation of lipase (6) and amylase (2). No treatment‐related deaths	57
dMMR/MSI‐H CRC	PD‐1 CTLA‐4	Nivolumab + ipilimumab CheckMate 142	2nd or more	II	Single arm	119	ORR, 55% (65 of 119), 4 CR, 61 PR	Grade 3 irAE in 32, AST or ALT (11%), lipase (4%), anemia or colitis (3%), hypothyroidism (1%). No treatment‐related deaths	58
HCC	PD‐1	Nivolumab, CheckMate 040	1st or more	I/II	Dose escalation and expansion	P I, 48 P II, 214	ORR, 15% (3 CR, 4 PR of 48) in P I, 20% in P II (3 CR, 39 PR of 214)	12 (25%) of 48 had grade 3/4 irAE; 3 (6%) had serious AE (PG, adrenal insufficiency, liver disorder)	59
PDAC	PD‐L1	BMS‐936559, CT00729664	1st or more	I	Dose escalation	Total, 207 PDAC, 16	PDAC, 0 of 14 (0%)	MTD was not reachedirAE, 81 of 207 (39%), included rash, hypothyroidism, hepatitis, diabetes mellitus	60

dMMR tumors^a^: 12 different tumor types.

AKI, acute kidney injury; BDS, bile duct stenosis; BO, bowel obstruction; CR, complete response; CRC, colorectal cancer; CTLA‐4, cytotoxic T‐lymphocyte‐associated protein 4; dMMR, defective mismatch repair; ESCC, esophageal squamous cell carcinoma; GC or GEJC, gastric or gastroesophageal junction cancer; HCC, hepatocellular carcinoma; IP, interstitial pneumonia; irAE, immune‐related adverse effect; Late, standard therapy failure; LFT, liver function test; MSI‐H, microsatellite instability‐high; MTD, maximum tolerated dose; Nivo, nivolumab; ORR, objective response rate; PD‐1, programmed cell death 1; PDAC, pancreatic ductal adenocarcinoma; PD‐L1, programmed cell death ligand 1; PE, pleural effusion; PG, pemphigoid; Place, placebo; pMMR, proficient mismatch repair; PR, partial response; PSN, peripheral sensory neuropathy; pts, patients; THR, thyroid hormone replacement.

**Table 3:** Approved immune checkpoint inhibitors for gastrointestinal cancers

Date	Target	Drug	Tumor type	Indication	Approval	Reference
22 Sep 17	PD‐1	Nivolumab	HCC	HCC previously treated with sorafenib	FDA	59
1 Aug 17	PD‐1	Nivolumab	CRC with MSI‐H or dMMR	MSI‐H or dMMR metastatic CRC that has progressed following treatment	FDA	57
22 Sep 17	PD‐1	Pembrolizumab	GC or GEJC	Previously treated patients with recurrent locally advanced or metastatic GC or GEJC whose tumors express PD‐L1	FDA	54
23 May 17	PD‐1	Pembrolizumab	Any solid tumor with dMMR or MSI‐H	First cancer treatment for any solid tumor with dMMR or MSI‐H	FDA	56
28 Sep 17	PD‐1	Nivolumab	GC or GEJC	Previously treated patients with advanced GC or GEJC	Japan	52

CRC, colorectal cancer; dMMR, defective mismatch repair; FDA, Food & Drug Administration; GC or GEJC, gastric or gastroesophageal junction cancer; HCC, hepatocellular carcinoma; MSI‐H, microsatellite instability‐high; PD‐1, programmed cell death 1; PD‐L1, programmed death ligand 1.

**Table 4:** Ongoing studies of immune checkpoint inhibitors

Tumor type	Target	Key drug	Trial design (arm)	Trial identifier	Treatment line	Phase	Allocation	Status	Sample size	Study start date	Estimated primary completion date
dMMR or MSI‐H CRC	PD‐1	Pembrolizumab	Pembrolizumab	Keynote 177	1st	III	Randomized	Active,	300	30 Nov 15	15 Aug 19
			Standard 1st‐line therapy for CRC	NCT02563002				not recruiting			
dMMR or MSI‐H CRC	PD‐1	Pembrolizumab	Pembrolizumab	Keynote 164	2nd or more	II	Single arm	Active,	124	25 Aug 15	09 Sep 19
				NCT02460198				not recruiting			
GC or GEJC	PD‐1	Nivolumab	Nivolumab + chemotherapy (SOX or CapeOX)	ONO‐4538‐37	1st	III	Randomized	Recruiting	680	Mar 16	Aug 20
			Placebo + chemotherapy (SOX or CapeOX)	NCT02746796							
GC or GEJC	PD‐1, CTLA‐4	Nivolumab + ipilimumab	Nivolumab + ipilimumab	CheckMate649	1st	III	Randomized	Recruiting	1349	04 Oct 16	12 Mar 20
			XELOX or FOLFOX	NCT02872116							
			Nivolumab + XELOX or FOLFOX								
GC	PD‐1	Nivolumab	Nivolumab + chemotherapy (S‐1 or CapeOX)	ONO‐4538‐38	adjuvant	III	Randomized	Recruiting	700	Jan 17	Jun 21
			Placebo + chemotherapy (S‐1 or CapeOX)	NCT03006705							
GC or GEJC	PD‐1	Pembrolizumab	Pembrolizumab monotherapy	KEYNOTE‐062	1st	III	Randomized	Active, not recruiting	764	31 Jul 15	05 Feb 19
			Pembrolizumab + cisplatin + 5‐FU	NCT02494583							
			Placebo + cisplatin + 5‐FU								
GC or GEJC	PD‐1	Pembrolizumab	Pembrolizumab	KEYNOTE‐063	2nd	III	Randomized	Active, not recruiting	360	16 Feb 17	17 Aug 19
			Paclitaxel	NCT03019588							
GC or GEJC	PD‐1	Pembrolizumab	Pembrolizumab + XP or FP	KEYNOTE‐585	Neoadjuvant	III	Randomized	Recruiting	860	09 Oct 17	26 Jul 23
			Placebo + XP or FP	NCT03221426	Adjuvant						
GC or GEJC	PD‐L1	Avelumab	Induction phase: FOLFOX or CapeOX	JAVELIN Gastric 100	1st	III	Randomized	Active, not recruiting	499	24 Dec 15	13 Mar 19
			Maintenance phase: avelumab	NCT02625610							
			Induction phase: FOLFOX or CapeOX								
			Maintenance phase: FOLFOX or CapeOX								
ESCC	PD‐1	Nivolumab	Nivolumab	ONO‐4538‐24	2nd	III	Randomized	Active, not recruiting	390	Dec 15	Sep 19
			Docetaxel or paclitaxel	NCT02569242							
ESCC	PD‐1	Nivolumab + ipilimumab	Nivolumab + ipilimumab	CheckMate 648	1st	III	Randomized	Recruiting	939	19 Jun 17	25 May 20
	CTLA‐4		Nivolumab + cisplatin + fluorouracil	NCT03143153							
			Cisplatin + fluorouracil								
ESCC	PD‐1	Pembrolizumab	Pembrolizumab	KEYNOTE‐181	2nd	III	Randomized	Recruiting	720	01 Dec 15	25 Sep 19
			Paclitaxel or docetaxel or irinotecan	NCT02564263							
EC or EGJC	PD‐1	Pembrolizumab	Pembrolizumab + cisplatin + 5‐FU	KEYNOTE‐590	1st	III	Randomized	Recruiting	700	25 Jul 17	22 Aug 21
			Placebo + cisplatin + 5‐FU	NCT03189719							
HCC	PD‐1	Nivolumab	Nivolumab	CheckMate 9DX	Adjuvant	III	Randomized	Recruiting	530	18 Dec 17	17 Apr 22
			Placebo	NCT03383458							
HCC	PD‐1	Nivolumab	Nivolumab	CheckMate 459	2nd	III	Randomized	Active, not recruiting	726	25 Nov 15	16 Oct 18
			Sorafenib	NCT02576509							
HCC	PD‐1	Pembrolizumab	Pembrolizumab + BSC	KEYNOTE‐394	2nd or more	III	Randomized	Recruiting	330	27 Apr 17	23 Dec 19
			Placebo + BSC	NCT03062358							
HCC	PD‐1	Pembrolizumab	Pembrolizumab + BSC	KEYNOTE‐240	2nd or more	III	Randomized	Active, not recruiting	408	26 May 16	01 Feb 19
			Placebo + BSC								

BSC, best supportive care; CRC, colorectal cancer; dMMR, defective mismatch repair; EC or EGJC, adenocarcinoma or squamous cell carcinoma of the esophagus or advanced/metastatic Siewert type 1 adenocarcinoma of the esophagogastric junction; ESCC, esophageal squamous cell carcinoma; FP, cisplatin + 5‐fluorouracil; GC or GEJC, gastric or gastroesophageal junction cancer; HCC, hepatocellular carcinoma; MSI‐H, microsatellite instability‐high; XP, cisplatin + capecitabine.

### 3.1 Esophageal cancer

Compared with other solid tumors, esophageal squamous cell carcinoma (ESCC) has a very high somatic mutation rate.^61^, ^62^ The high mutation load in esophageal tumors has been associated with the clinical benefit of PD‐1 blockade.^63^ Nivolumab is a human monoclonal immunoglobulin (Ig)G4 antibody that seals PD‐1 expressed on activated T cells. This drug was applied to treatment‐refractory esophageal cancer in an open‐label, multicenter, phase II trial (Table 2). Nivolumab showed promising activity with a manageable safety profile.^51^ PD‐1/PD‐L1 blockade alone or in combination with radiotherapy and chemotherapy will be a direction for future research in the treatment of advanced esophageal cancer (Table 4).

### 3.2 Gastric cancer

To assess the efficacy and safety of nivolumab in patients with advanced gastric cancer (GC) or gastroesophageal junction cancer (GEJC) refractory to, or intolerant of, two or more previous regimens of chemotherapy, a randomized, double‐blind, placebo‐controlled, phase III trial was carried out (Table 2). In that phase III study, survival benefits indicated that nivolumab might represent a new treatment option for heavily pretreated patients with advanced GC or GEJC. Based on that study, nivolumab was approved in Japan for unresectable advanced or recurrent gastric cancer that has progressed after chemotherapy (Table 3).^52^

Pembrolizumab uses another developmental strategy to target patients with programmed death ligand 1 (PD‐L1)‐positive advanced gastric cancer. A phase Ib trial designed to assess the safety and activity of pembrolizumab was carried out in patients with PD‐L1‐positive recurrent or metastatic GC or GEJC. Patients received i.v. pembrolizumab at 10 mg/kg once every 2 weeks (Table 2). Pembrolizumab showed a manageable toxicity profile and promising antitumor activity.^53^ The FDA approved pembrolizumab for previously treated patients with recurrent locally advanced or metastatic GC or GEJC whose tumors express PD‐L1. This decision was based on data from a global multicohort trial, KEYNOTE‐059, which indicated a superior response in patients with tumors that expressed PD‐L1 (Tables 2,3).^54^

Ongoing trials are investigating various settings and earlier treatment lines for GC or GEJC (Table 4).

### 3.3 Colorectal cancer

In early‐phase studies, responses of CRC to PD‐1/PD‐L1 inhibitors were not promising.^60^ No objective response was seen in patients treated with PD‐1 inhibitors (0/19)^49^ or PD‐L1 inhibitors (0/16). Surprisingly, updated reports have indicated that patients with metastatic CRC who harbored the microsatellite instability‐high (MSI‐H) genotype achieved objective responses after disease progression on an intermittent dosing regimen of PD‐1 inhibitors and finally achieved complete responses.^50^ The majority of colorectal cancers are proficient mismatch repair (pMMR) tumors, and approximately 15% show defective mismatch repair (dMMR), which can be measured by either the five‐marker panel with fluorescent multiplex assay^64^ or by the lack of DNA mismatch repair proteins.^65^ Tumors with dMMR can have MSI‐H and a somatic mutation frequency of more than 10‐ to 100‐fold that of pMMR tumors.^66^ Hence, dMMR (MSI‐H) tumor is thought to have the potential to encode “non‐self” immunogenic antigens and predict responsiveness to the immune checkpoint blockade (Tables 2, 3, 4).

Based on this perspective, a phase II study was conducted to evaluate the clinical activity of pembrolizumab, an anti‐PD‐1 immune checkpoint inhibitor, and 32 patients with progressive metastatic carcinoma with or without dMMR were enrolled and received i.v. pembrolizumab. Objective response rates (ORR) were 40% for dMMR colorectal cancers and 0% for pMMR colorectal cancers. Median PFS and OS were not reached in the cohort with dMMR CRC, but were 2.2 months and 5.0 months, respectively, in the cohort with pMMR CRC. Whole‐exome sequencing showed a mean of 1782 somatic mutations per tumor in dMMR tumors, as compared with 73 in pMMR tumors, and high somatic mutation loads were associated with prolonged PFS (*P* = .02). This study showed that patients with dMMR are good candidates for receiving immune checkpoint blockade (Table 2).^55^

Next, this study was expanded to evaluate the efficacy of PD‐1 blockade in patients with advanced dMMR cancers across 12 different tumor types. Responses were durable with median PFS and OS still not reached. These data support the hypothesis that the large proportion of mutant neoantigens in dMMR cancers make them sensitive to immune checkpoint blockade, regardless of the tissue of origin for the cancer,^56^ and the FDA approved the use of pembrolizumab in the treatment of patients with MSI‐H or dMMR (Tables 2, 3).

Similar results were obtained from an open‐label, phase II study of nivolumab in patients with dMMR/MSI‐H metastatic CRC. Patients were given nivolumab at 3 mg/kg every 2 weeks until disease progression or unacceptable toxic effects (Table 2).^57^ The FDA approved nivolumab use in the treatment for MSI‐H or dMMR metastatic colorectal cancer that has progressed following treatment (Table 3). Studies of immune checkpoint inhibitors are ongoing to indicate potential efficacy as first‐line agents (Table 4).

### 3.4 Hepatocellular carcinoma

The only evidence‐based systemic treatment option is sorafenib, a small‐molecule multikinase inhibitor, for patients with advanced hepatocellular carcinoma (HCC).^67^ The presence of tumor‐infiltrating lymphocytes expressing PD‐1 in HCC lesions and their correlation with outcome suggest that immunotherapeutic approaches might be useful in this setting.^68^ To assess the safety and efficacy of nivolumab, a phase I/II dose escalation and expansion trial was designed in patients with advanced HCC with or without chronic viral hepatitis. Eligible patients had Child‐Pugh scores of 7 or less (Child‐Pugh A or B7) for the dose‐escalation phase and 6 or less (Child‐Pugh A) for the dose‐expansion phase. Patients with hepatitis B virus (HBV) infection had to be receiving effective antiviral therapy (viral load <100 IU/mL), but antiviral therapy was not required for patients with hepatitis C virus (HCV) infection. Patients received i.v. nivolumab at 0.1‐10 mg/kg every 2 weeks in the dose‐escalation phase (3 + 3 design). Nivolumab 3 mg/kg was given every 2 weeks in the dose‐expansion phase to patients in four cohorts: sorafenib untreated or intolerant without viral hepatitis; sorafenib failure without viral hepatitis; HCV infected; and HBV infected. A total of 262 eligible patients were treated. During dose escalation, nivolumab showed a manageable safety profile, including acceptable tolerability. Incidence of treatment‐related adverse events did not seem to be associated with dose and no maximum tolerated dose was reached, and nivolumab 3 mg/kg was chosen for dose expansion. Durable objective responses show the potential for nivolumab in the treatment of advanced HCC (Table 2),^59^ and nivolumab received FDA approval for the treatment of hepatocellular carcinoma patients previously treated with sorafenib (Table 3).

### 3.5 Pancreatic ductal adenocarcinoma

Pancreatic ductal adenocarcinoma (PDAC) is one of the most lethal malignancies worldwide. Desmoplasia (abundant fibrotic stroma) is a typical feature of PDAC in humans, and stromal activation commonly starts around precancerous lesions. Cancer‐stroma interactions affect tumorigenesis, angiogenesis, therapy resistance and, possibly, the metastatic spread of tumor cells.^69^ In fact, no objective response was observed in 14 pancreatic cancer (PC) patients treated with BMS‐936559, an anti‐PD‐L1 antibody (Table 2).^60^ Therefore, targeting the tumor stroma offers a promising new option for the treatment of PDAC. The immunosuppressive environment surrounding PDAC appears to be one of the major obstacles to the development of successful therapies for this fatal disease.^69^

## 4 COMBINATION IMMUNOTHERAPY BASED ON PRECISION MEDICINE: BEYOND IMMUNE CHECKPOINT INHIBITORS

Digestive cancers are tough targets for immunotherapy. ORR of PD‐1/PD‐L1 blockade were 17% for ESCC,^51^ 11.2% for GC,^52^, ^54^ 20% for HCC,^59^ and 0% for PDAC.^60^ dMMR tumors can be MSI‐H and show a somatic mutation frequency of more than 10‐100‐fold those of pMMR/microsatellite‐stable (MSS) tumors.^66^ Hence, dMMR/MSI‐H tumor is thought to have the potential to encode “non‐self” immunogenic antigens and is thought to respond well to immune checkpoint blockade. An interesting phase II study was evaluated for the clinical activity of pembrolizumab, an anti‐PD‐1 inhibitor. ORR was 40% for dMMR CRC, 71% for dMMR non‐CRC, and 0% for pMMR CRC.^55^ Blockade of PD‐1/PD‐L1 signaling brings durable efficacy for a subset of patients showing high tumor mutation burden (TMB) across almost all types of cancer, including many rare tumor types, characterized by dMMR/MSI‐H.^62^ The combination of nivolumab (PD‐1 blocker) plus ipilimumab (CTLA‐4 blocker) in dMMR/MSI‐H CRC was recently reported. Patients had an ORR of 55% and a DCR of 80%.^58^ However, not all patients with dMMR/MSI‐H achieved cure, even with the combination of immune checkpoint inhibitors, because of insufficient neoantigens, high tumor burden, suppressive immunity of the tumor microenvironment, or exhaustion of systemic immunosurveillance.^70^ These results clearly indicate that suppressive immunity should be controlled using a multidisciplinary approach (Table 5). PD‐L1 expression, CD8^+^ tumor‐infiltrating lymphocytes, and tumor mutation load are well‐known biomarkers of immune checkpoint inhibitors.^86^, ^87^

**Table 5:** List of suppressive immunity and its resolution methods

Suppressive immunity	Therapeutic strategies	References
Treg	Cyclophosphamide, metformin	71, 72, 73, 74
MDSC	COX2 inhibitors, cimetidine	71, 75, 76
TIM‐3 expression on T cells (immune exhaustion)	Metformin, poly(I:C) plus LAG‐3‐Ig	44, 77
IL‐6	COX2 inhibitors, anti‐IL‐6 antibody	78, 79
TGF‐β	TGF‐β receptor inhibitor	80
PGE2	COX2 inhibitors	71, 81
CAF	FAK inhibition	82
TAM	TGF‐β‐activated kinase‐1 inhibitor	83
Poor CTL infiltration	Cetuximab, activate CD4^+^ lymphocytes, cancer antigen‐specific immunotherapy	17, 46, 47, 48, 84
PD‐1 expression on T cells	PD‐1 blockade, poly(I:C) plus LAG‐3‐Ig	44, 49
PD‐L1 expression on tumor cells	PD‐1/PD‐L1 blockade	49, 60
IDO	IDO inhibitor	85

CAF, cancer‐associated fibroblast; COX, cyclooxygenase; FAK, focal adhesion kinase; IDO, indoleamine‐2,3‐dioxygenase; Ig, immunoglobulin; IL‐6, interleukin‐6; LAG, lymphocyte activation gene; MDSC, myeloid‐derived suppressor cell; PD‐1, programmed cell death 1; PD‐L1, programmed cell death ligand 1; PGE2, prostaglandin E2; TAM, tumor‐associated macrophage; TGF, transforming growth factor; TIM‐3, T‐cell immunoglobulin and mucin domain‐containing protein‐3; Treg, regulatory T cell.

**Figure 1:**
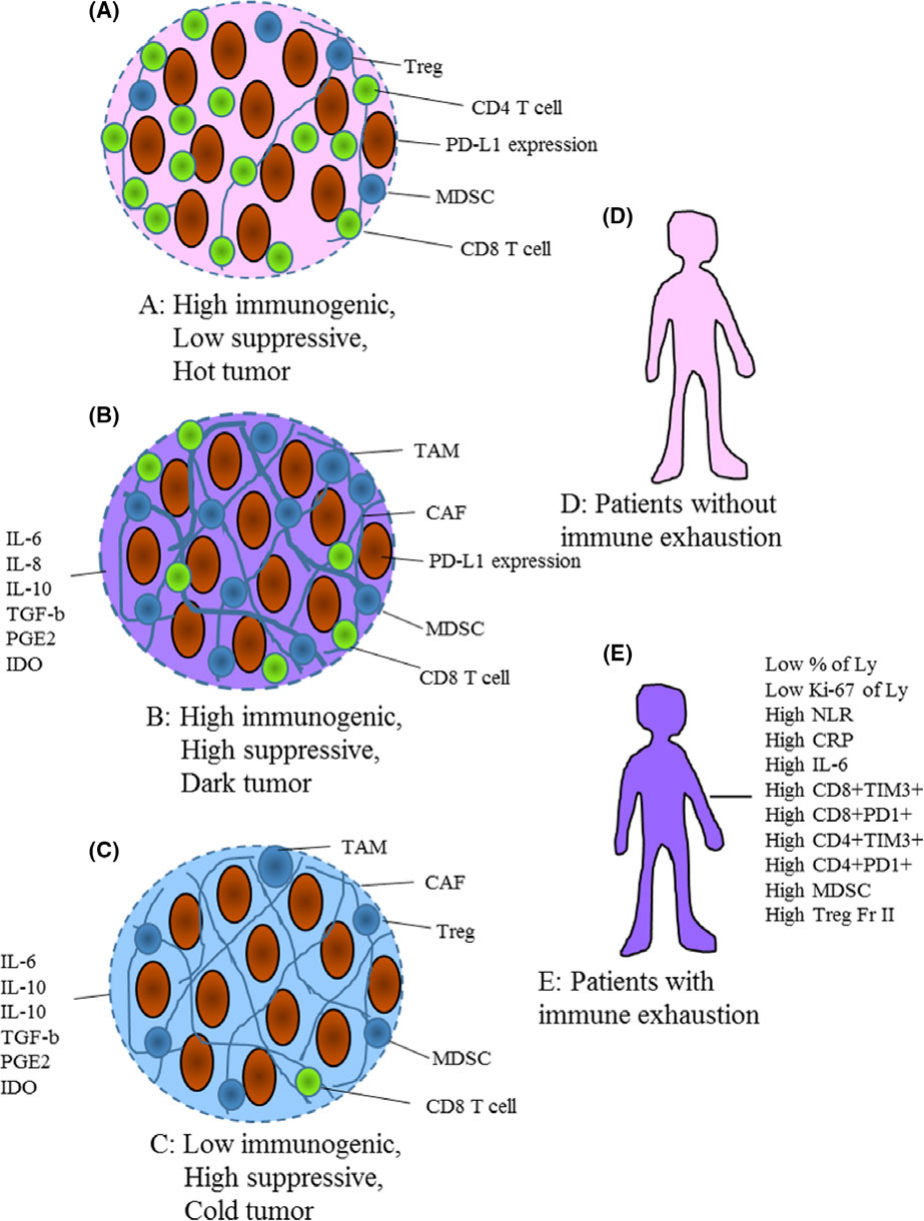
Concept of immunological status of various tumors or patients and implications for immunotherapy. A, Hot tumor might respond well to immune checkpoint inhibitors. B, Dark tumor might need immune checkpoint inhibitors and agents to resolve suppressive immunity. C, Cold tumor might require combination therapies comprising an agent to create an immunogenic tumor microenvironment plus immune checkpoint inhibitors and agents to resolve suppressive immunity. D, Patients without immune exhaustion might not need additional treatment. E, Patients with immune exhaustion might need additional treatment to resolve exhaustion. CAF, cancer‐associated fibroblasts; CRP, C‐reactive protein; IDO, indoleamine‐2,3‐dioxygenase; IL, interleukin; MDSC, myeloid‐derived suppressor cells; NLR, neutrophil‐to‐lymphocyte ratio; PD‐1, programmed cell death 1; PD‐L1, programmed death ligand 1; PGE2, prostaglandin E2; TAM, tumor‐associated macrophage; TGF‐β, transforming growth factor beta; TIM‐3, T‐cell immunoglobulin and mucin domain‐containing protein‐3; Treg, regulatory T cell

Recent immune checkpoint blockade therapy has basically shown two mechanisms of immunosuppression. First, through the production of secreted suppressive molecules such as transforming growth factor (TGF)‐β, IL‐6, and prostaglandin E2 (PGE2) and, second, through various immunosuppressive cells such as regulatory T cells (Treg), myeloid‐derived suppressor cells (MDSC), tumor‐associated macrophages (TAM), and cancer‐associated fibroblasts (CAF), which are partly induced by the secreted factors mentioned above (Figure 1A‐C). These molecules and cells are supplied to lymph nodes where anti‐tumor T cells are induced and are subsequently immunologically suppressed in patients with cancer (Figure 1D,E).^8^

One way to achieve successful immunotherapies is to establish biomarkers for excluding those patients unlikely to respond to immunotherapy (Figure 1E). The old‐new biomarker is NLR, which is prognostic in many oncological settings. NLR kinetics in patients with advanced solid tumors treated with PD‐1/PD‐L1 inhibitors showed that the median OS for patients with a high NLR was 8.5 months, compared to 19.4 for patients with low NLR (*P* = .01).^88^ The importance of NLR was also reported in a study of a peptide vaccine against CRC.^17^ Circulating CD8^+^ T cells are incontrovertibly important. Huang et al^70^ reported that the T‐cell invigoration‐to‐tumor burden ratio was associated with anti‐PD‐1 response, and T‐cell invigoration could be monitored as low expression of Ki‐67 on circulating CD8 T cells. To alter the T‐cell invigoration‐to‐tumor burden ratio, chemotherapy or radiation might be useful strategies in combination with immune checkpoint inhibitors, and numerous phase studies of such combination therapy are ongoing.

Another way to achieve successful immunotherapies is to alter tumor microenvironments and host immunosurveillance. So‐called “hot tumor” with massive infiltration of CD8^+^ cells and without suppressive immunity could respond well to immune checkpoint inhibitor alone (Figure 1A,D). Each of “dark tumor” (Figure 1B), which is highly immunogenic but with suppressive immunity, “cold tumor” (Figure 1C), which shows low immunogenicity with suppressive immunity, and patients with immune exhaustion (Figure 1E) might require a multidisciplinary approach (Table 5).

The immunosuppressive environment surrounding PDAC might be one of the major obstacles to the development of successful therapies for this fatal disease.^69^ Advances in our understanding of the immunosuppressive mechanisms in PDAC might lead to promising immunotherapeutic approaches. PDAC patients displayed an increased number of Treg and MDSC.^71^ Cyclophosphamide^72^ and metformin^73^ could downregulate the number and function of Treg, and cyclooxygenase (COX)‐2 inhibitors^75^ and cimetidine^76^ could regulate MDSC. Metformin is also reported to have a direct effect on CD8^+^ T cells for protection against the inevitable functional exhaustion marker T‐cell immunoglobulin and mucin domain‐containing protein‐3 (Tim‐3) in the tumor microenvironment.^77^ The production of secreted suppressive molecules such as IL‐6 leads to the limited efficacy of immune checkpoint inhibitors in PDAC, so IL‐6 blockade would modulate the immunological features of PDAC. An experimental study of combined IL‐6 and PD‐L1 blockade elicited efficacy in mice bearing s.c. tumors, accompanied by increased intratumoral effector T lymphocytes. These preclinical results indicate that targeted inhibition of IL‐6 may enhance the efficacy of anti‐PD‐L1 in PDAC (Table 5).^78^ Other inhibitory factors in the tumor microenvironment, such as TGF‐β^80^ and PGE2^75^, ^81^ and its inhibitors, are summarized in Table 5.

Another problem is the presence of a uniquely desmoplastic stroma that functions as a barrier to T‐cell infiltration (Figure 1B,C). Hyperactivated focal adhesion kinase (FAK) activity in PDAC cells is an important regulator of the fibrotic and immunosuppressive microenvironments. FAK activity is elevated in human PDAC tissues and correlates with high levels of fibrosis and poor CD8^+^ cytotoxic T‐cell infiltration.^82^ Single‐agent FAK inhibition using the selective FAK inhibitor VS‐4718 substantially limited tumor progression, resulting in a doubling of survival in a mouse model of human PDAC. This delay in tumor progression was associated with markedly reduced CAF and decreased numbers of tumor‐infiltrating immunosuppressive cells.^82^ TAM^83^ are also an inhibitory factor in tumor microenvironments, and associated therapeutic strategies are shown in Table 5.

Tumor tissues that lack expression of many immunological markers may indicate a non‐immunogenic tumor microenvironment (Figure 1C), which may require combination therapies consisting of an agent to create an immunogenic tumor microenvironment plus an immune checkpoint agent to further enhance immune responses for clinical benefit.^86^ Conventional cancer therapies such as chemotherapy or radiation may also lead to tumor cell death and release of antigens to initiate activation of T cells, which may then migrate into tumor tissues. Combination studies using conventional agents and immune checkpoint therapies should thus clarify the conditions needed to create an “immunogenic” tumor microenvironment with subsequent clinical benefit for patients.^86^ We have reported that cetuximab strongly enhances immune cell infiltration into liver metastatic sites in CRC.^46^ Cetuximab induces antibody‐dependent cell‐mediated cytotoxicity and immunogenic cell death. We assessed immune cell infiltration into liver metastatic sites of 53 CRC patients treated with chemotherapy and cetuximab, chemotherapy without cetuximab, and no chemotherapy. Of note, inflammatory cells were found in intratumoral areas, and the destruction of cancer cell foci was observed in the cetuximab group. Moreover, higher infiltration of CD8^+^ (*P* = .003) and CD56^+^ (*P* = .001) cells was observed in the cetuximab group. The immune‐related mechanism of cetuximab may enhance the efficacy of combination therapy using immune checkpoint inhibitors and/or therapeutic peptides.^46^

Although no objective responder to pembrolizumab is seen in pMMR/MSS‐CRC, some patients obtained SD lasting more than 3 months according to tumor markers as well as radiographical evaluation,^55^ indicating that some groups of MSS‐CRC could respond to PD‐1 blockade. Galon et al^89^ attested that MSS colon cancer is divided into tumors with or without massive CD8^+^ T‐cell infiltration. The reason why metastatic MSS‐CRC did not respond well to immune checkpoint inhibitors might be divided into two patterns, owing to the highly immunosuppressive state of the “dark tumor” microenvironment (Figure 1B) and the low immunogenicity in the “cold tumor” microenvironment (Figure 1C). For dark tumors, a combination of immune checkpoint inhibitors and the modalities summarized in Table 5 to resolve the suppressive immunity might prove effective. For cold tumors (Figure 1C), additional use of methods to induce immune cells to the tumor site would be needed, as described above. The combination of neoantigen‐derived vaccination and cetuximab might be one of the most promising strategies.

Hence, combination immunotherapy should be selected as a precision medicine based on comprehensive analyses using whole‐exome sequencing and RNA sequences. Moreover, novel immune checkpoints might not yet have been detected. Absolutely effective combination strategies might be just around the corner.

## DISCLOSURE

Funding: This study was carried out as a research program of the Project for Development of Innovative Research on Cancer Therapeutics (P‐DIRECT; 11039020), The Japan Agency for Medical Research and Development (AMED; 15cm0106085 h0005), and this study was supported in part by a grant for Leading Advanced Projects for Medical Innovation (LEAP; 16am0001006 h0003) from the Japan Agency for Medical Research and Development.Conflicts of Interest: Shoichi Hazama received research funding from NEC Corporation and Toyo Kohan Corporation. Koji Tamada has a leadership role in, and owns stock of, Noile‐ImmuneBioteck, Inc., and received research funding from Noile‐ImmuneBioteck, Inc. and NEC Corporation. Yoshiyuki Yamaguchi received research funding from Chugai Pharmaceutical Company, Yakult Honsha Company, Kyowa Hakko Kirin Company, Takeda Pharmaceutical Company, Daiichi Sankyo Healthcare Company, Ono Pharmaceutical Company, Taiho Pharmaceutical Company, and Bristol‐Myers Squibb, and lecture fees from Chugai Pharmaceutical Company, Ono Pharmaceutical Company. Yutaka Kawakami received research funding and lecture fees and manuscript payments from Ono Pharmaceutical Company and Bristol‐Myers Squibb.

## References

[1] Ferlay J , Soerjomataram I , Dikshit R , et al. Cancer incidence and mortality worldwide: sources, methods and major patterns in GLOBOCAN 2012. Int J Cancer. 2015;136:E359–86. 25220842 10.1002/ijc.29210

[2] Long J , Lin J , Wang A , et al. PD‐1/PD‐L blockade in gastrointestinal cancers: lessons learned and the road toward precision immunotherapy. J Hematol Oncol. 2017;10:146. 28774337 10.1186/s13045-017-0511-2 PMC5543600

[3] Myint ZW , Goel G . Role of modern immunotherapy in gastrointestinal malignancies: a review of current clinical progress. J Hematol Oncol. 2017;10:86. 28434400 10.1186/s13045-017-0454-7 PMC5402172

[4] Coley WB II . Contribution to the Knowledge of Sarcoma. Ann Surg. 1891;14:199–220. 10.1097/00000658-189112000-00015 PMC1428624 17859590

[5] Smith T . Active Immunity Produced by So Called Balanced or Neutral Mixtures of Diphtheria Toxin and Antitoxin. J Exp Med. 1909;11:241–56. 19867246 10.1084/jem.11.2.241 PMC2124709

[6] Burnet FM . The concept of immunological surveillance. Prog Exp Tumor Res. 1970;13:1–27. 4921480 10.1159/000386035

[7] van der Bruggen P , Traversari C , Chomez P , et al. A gene encoding an antigen recognized by cytolytic T lymphocytes on a human melanoma. Science. 1991;254:1643–7. 1840703 10.1126/science.1840703

[8] Yaguchi T , Kawakami Y . Cancer‐induced heterogeneous immunosuppressive tumor microenvironments and their personalized modulation. Int Immunol. 2016;28:393–9. 27401477 10.1093/intimm/dxw030 PMC4986236

[9] Oba MS , Teramukai S , Ohashi Y , Ogawa K , Maehara Y , Sakamoto J . The efficacy of adjuvant immunochemotherapy with OK‐432 after curative resection of gastric cancer: an individual patient data meta‐analysis of randomized controlled trials. Gastric Cancer. 2016;19:616–24. 25804300 10.1007/s10120-015-0489-9

[10] Yoshino S , Nishikawa K , Morita S , et al. Randomised phase III study of S‐1 alone versus S‐1 plus lentinan for unresectable or recurrent gastric cancer (JFMC36‐0701). Eur J Cancer. 2016;65:164–71. 27501505 10.1016/j.ejca.2016.06.012

[11] Oba K , Teramukai S , Kobayashi M , Matsui T , Kodera Y , Sakamoto J . Efficacy of adjuvant immunochemotherapy with polysaccharide K for patients with curative resections of gastric cancer. Cancer Immunol Immunother. 2007;56:905–11. 17106715 10.1007/s00262-006-0248-1 PMC11030720

[12] Rayner AA , Grimm EA , Lotze MT , Chu EW , Rosenberg SA . Lymphokine‐activated killer (LAK) cells. Analysis of factors relevant to the immunotherapy of human cancer. Cancer. 1985;55:1327–33. 3871657 10.1002/1097-0142(19850315)55:6<1327::aid-cncr2820550628>3.0.co;2-o

[13] Turcotte S , Gros A , Tran E , et al. Tumor‐reactive CD8+ T cells in metastatic gastrointestinal cancer refractory to chemotherapy. Clin Cancer Res. 2014;20:331–43. 24218514 10.1158/1078-0432.CCR-13-1736 PMC3927404

[14] Maeda Y , Yoshimura K , Matsui H , et al. Dendritic cells transfected with heat‐shock protein 70 messenger RNA for patients with hepatitis C virus‐related hepatocellular carcinoma: a phase 1 dose escalation clinical trial. Cancer Immunol Immunother. 2015;64:1047–56. 25982372 10.1007/s00262-015-1709-1 PMC11028566

[15] Shindo Y , Hazama S , Maeda Y , et al. Adoptive immunotherapy with MUC1‐mRNA transfected dendritic cells and cytotoxic lymphocytes plus gemcitabine for unresectable pancreatic cancer. J Transl Med. 2014;12:175. 24947606 10.1186/1479-5876-12-175 PMC4074851

[16] Yamada A , Sasada T , Noguchi M , Itoh K . Next‐generation peptide vaccines for advanced cancer. Cancer Sci. 2013;104:15–21. 23107418 10.1111/cas.12050 PMC7657262

[17] Hazama S , Nakamura Y , Tanaka H , et al. A phase II study of five peptides combination with oxaliplatin‐based chemotherapy as a first‐line therapy for advanced colorectal cancer (FXV study). J Transl Med. 2014;12:108. 24884643 10.1186/1479-5876-12-108 PMC4021539

[18] Miyazawa M , Katsuda M , Maguchi H , et al. Phase II clinical trial using novel peptide cocktail vaccine as a postoperative adjuvant treatment for surgically resected pancreatic cancer patients. Int J Cancer. 2017;140:973–82. 27861852 10.1002/ijc.30510

[19] Shimizu Y , Suzuki T , Yoshikawa T , et al. Cancer immunotherapy targeted glypican‐3 or neoantigens. Cancer Sci. 2018;109:531–41. 29285841 10.1111/cas.13485 PMC5834776

[20] Nagorsen D , Thiel E . Clinical and immunologic responses to active specific cancer vaccines in human colorectal cancer. Clin Cancer Res. 2006;12:3064–9. 16707603 10.1158/1078-0432.CCR-05-2788

[21] Couzin‐Frankel J . Breakthrough of the year 2013. Cancer Immunotherapy. Science. 2013;342:1432–3. 24357284 10.1126/science.342.6165.1432

[22] Mule JJ , Shu S , Schwarz SL , Rosenberg SA . Adoptive immunotherapy of established pulmonary metastases with LAK cells and recombinant interleukin‐2. Science. 1984;225:1487–9. 6332379 10.1126/science.6332379

[23] Takayama T , Sekine T , Makuuchi M , et al. Adoptive immunotherapy to lower postsurgical recurrence rates of hepatocellular carcinoma: a randomised trial. Lancet. 2000;356:802–7. 11022927 10.1016/S0140-6736(00)02654-4

[24] Kaufmann Y , Moscovitch M , Robb RJ , Rosenberg SA , Berke G . Antigen/mitogen induced cytolytic activity and IL‐2 secretion in memory‐like CTL‐hybridomas. Adv Exp Med Biol. 1985;184:535–50. 3929574 10.1007/978-1-4684-8326-0_35

[25] Matsui H , Hazama S , Sakamoto K , et al. Postoperative adjuvant therapy for resectable pancreatic cancer with gemcitabine and adoptive immunotherapy. Pancreas. 2017;46:994–1002. 28697053 10.1097/MPA.0000000000000880 PMC5555975

[26] Hazama S , Nakamura Y , Takenouchi H , et al. A phase I study of combination vaccine treatment of five therapeutic epitope‐peptides for metastatic colorectal cancer; safety, immunological response, and clinical outcome. J Transl Med. 2014;12:63. 24612787 10.1186/1479-5876-12-63 PMC4007571

[27] Sawada Y , Yoshikawa T , Ofuji K , et al. Phase II study of the GPC3‐derived peptide vaccine as an adjuvant therapy for hepatocellular carcinoma patients. Oncoimmunology. 2016;5:e1129483. 27467945 10.1080/2162402X.2015.1129483 PMC4910752

[28] Rosenberg SA . The development of new immunotherapies for the treatment of cancer using interleukin‐2. A review. Ann Surg. 1988;208:121–35. 3041925 10.1097/00000658-198808000-00001 PMC1493624

[29] Aruga A , Yamauchi K , Takasaki K , Furukawa T , Hanyu F . Induction of autologous tumor‐specific cytotoxic T cells in patients with liver cancer. Characterizations and clinical utilization. Int J Cancer. 1991;49:19–24. 1678733 10.1002/ijc.2910490105

[30] Kawaoka T , Oka M , Takashima M , et al. Adoptive immunotherapy for pancreatic cancer: cytotoxic T lymphocytes stimulated by the MUC1‐expressing human pancreatic cancer cell line YPK‐1. Oncol Rep. 2008;20:155–63. 18575732

[31] June CH , O'Connor RS , Kawalekar OU , Ghassemi S , Milone MC . CAR T cell immunotherapy for human cancer. Science. 2018;359:1361–5. 29567707 10.1126/science.aar6711

[32] Porter DL , Levine BL , Kalos M , Bagg A , June CH . Chimeric antigen receptor‐modified T cells in chronic lymphoid leukemia. N Engl J Med. 2011;365:725–33. 21830940 10.1056/NEJMoa1103849 PMC3387277

[33] Chen C , Li K , Jiang H , et al. Development of T cells carrying two complementary chimeric antigen receptors against glypican‐3 and asialoglycoprotein receptor 1 for the treatment of hepatocellular carcinoma. Cancer Immunol Immunother. 2017;66:475–89. 28035433 10.1007/s00262-016-1949-8 PMC11028818

[34] Zhang C , Wang Z , Yang Z , et al. Phase I escalating‐dose trial of CAR‐T therapy targeting CEA(+) metastatic colorectal cancers. Mol Ther. 2017;25:1248–58. 28366766 10.1016/j.ymthe.2017.03.010 PMC5417843

[35] Feng K , Liu Y , Guo Y , et al. Phase I study of chimeric antigen receptor modified T cells in treating HER2‐positive advanced biliary tract cancers and pancreatic cancers. Protein Cell. 2017; https://doi.org/10.1007/s13238-017-0440-4 10.1007/s13238-017-0440-4 PMC6160389 28710747

[36] Adachi K , Kano Y , Nagai T , Okuyama N , Sakoda Y , Tamada K . IL‐7 and CCL19 expression in CAR‐T cells improves immune cell infiltration and CAR‐T cell survival in the tumor. Nat Biotechnol. 2018;36:346–51. 29505028 10.1038/nbt.4086

[37] Nestle FO , Alijagic S , Gilliet M , et al. Vaccination of melanoma patients with peptide‐ or tumor lysate‐pulsed dendritic cells. Nat Med. 1998;4:328–32. 9500607 10.1038/nm0398-328

[38] Yoshida S , Hazama S , Tokuno K , et al. Concomitant overexpression of heat‐shock protein 70 and HLA class‐I in hepatitis C virus‐related hepatocellular carcinoma. Anticancer Res. 2009;29:539–44. 19331200

[39] Eura M , Chikamatsu K , Ogi K , Nakano K , Masuyama K , Ishikawa T . Expression of genes MAGE‐1, ‐2, and ‐3 by human maxillary carcinoma cells. Anticancer Res. 1995;15:55–9. 7733641

[40] Sawada Y , Yoshikawa T , Nobuoka D , et al. Phase I trial of a glypican‐3‐derived peptide vaccine for advanced hepatocellular carcinoma: immunologic evidence and potential for improving overall survival. Clin Cancer Res. 2012;18:3686–96. 22577059 10.1158/1078-0432.CCR-11-3044

[41] Sawada Y , Yoshikawa T , Fujii S , et al. Remarkable tumor lysis in a hepatocellular carcinoma patient immediately following glypican‐3‐derived peptide vaccination: an autopsy case. Hum Vaccin Immunother. 2013;9:1228–33. 23466818 10.4161/hv.24179 PMC3901810

[42] Rosenberg SA , Yang JC , Restifo NP . Cancer immunotherapy: moving beyond current vaccines. Nat Med. 2004;10:909–15. 15340416 10.1038/nm1100 PMC1435696

[43] Marin‐Acevedo JA , Soyano AE , Dholaria B , Knutson KL , Lou Y . Cancer immunotherapy beyond immune checkpoint inhibitors. J Hematol Oncol. 2018;11:8. 29329556 10.1186/s13045-017-0552-6 PMC5767051

[44] Kano Y , Iguchi T , Matsui H , et al. Combined adjuvants of poly(I:C) plus LAG‐3‐Ig improve antitumor effects of tumor‐specific T cells, preventing their exhaustion. Cancer Sci. 2016;107:398–406. 27079438 10.1111/cas.12861 PMC4832865

[45] Gobel C , Breitenbuecher F , Kalkavan H , et al. Functional expression cloning identifies COX‐2 as a suppressor of antigen‐specific cancer immunity. Cell Death Dis. 2014;5:e1568. 25501829 10.1038/cddis.2014.531 PMC4649842

[46] Inoue Y , Hazama S , Suzuki N , et al. Cetuximab strongly enhances immune cell infiltration into liver metastatic sites in colorectal cancer. Cancer Sci. 2017;108:455–60. 28075526 10.1111/cas.13162 PMC5378263

[47] Ott PA , Hu Z , Keskin DB , et al. An immunogenic personal neoantigen vaccine for patients with melanoma. Nature. 2017;547:217–21. 28678778 10.1038/nature22991 PMC5577644

[48] Sahin U , Derhovanessian E , Miller M , et al. Personalized RNA mutanome vaccines mobilize poly‐specific therapeutic immunity against cancer. Nature. 2017;547:222–6. 28678784 10.1038/nature23003

[49] Topalian SL , Hodi FS , Brahmer JR , et al. Safety, activity, and immune correlates of anti‐PD‐1 antibody in cancer. N Engl J Med. 2012;366:2443–54. 22658127 10.1056/NEJMoa1200690 PMC3544539

[50] Lipson EJ , Sharfman WH , Drake CG , et al. Durable cancer regression off‐treatment and effective reinduction therapy with an anti‐PD‐1 antibody. Clin Cancer Res. 2013;19:462–8. 23169436 10.1158/1078-0432.CCR-12-2625 PMC3548952

[51] Kudo T , Hamamoto Y , Kato K , et al. Nivolumab treatment for oesophageal squamous‐cell carcinoma: an open‐label, multicentre, phase 2 trial. Lancet Oncol. 2017;18:631–9. 28314688 10.1016/S1470-2045(17)30181-X

[52] Kang YK , Boku N , Satoh T , et al. Nivolumab in patients with advanced gastric or gastro‐oesophageal junction cancer refractory to, or intolerant of, at least two previous chemotherapy regimens (ONO‐4538‐12, ATTRACTION‐2): a randomised, double‐blind, placebo‐controlled, phase 3 trial. Lancet. 2017;390:2461–71. 28993052 10.1016/S0140-6736(17)31827-5

[53] Muro K , Chung HC , Shankaran V , et al. Pembrolizumab for patients with PD‐L1‐positive advanced gastric cancer (KEYNOTE‐012): a multicentre, open‐label, phase 1b trial. Lancet Oncol. 2016;17:717–26. 27157491 10.1016/S1470-2045(16)00175-3

[54] Fuchs CS , Doi T , Jang RW‐J , et al. KEYNOTE‐059 cohort 1: efficacy and safety of pembrolizumab (pembro) monotherapy in patients with previously treated advanced gastric cancer. J Clin Oncol. 2017;35:4003.29040031

[55] Le DT , Uram JN , Wang H , et al. PD‐1 blockade in tumors with mismatch‐repair deficiency. N Engl J Med. 2015;372:2509–20. 26028255 10.1056/NEJMoa1500596 PMC4481136

[56] Le DT , Durham JN , Smith KN , et al. Mismatch repair deficiency predicts response of solid tumors to PD‐1 blockade. Science. 2017;357:409–13. 28596308 10.1126/science.aan6733 PMC5576142

[57] Overman MJ , McDermott R , Leach JL , et al. Nivolumab in patients with metastatic DNA mismatch repair‐deficient or microsatellite instability‐high colorectal cancer (CheckMate 142): an open‐label, multicentre, phase 2 study. Lancet Oncol. 2017;18:1182–91. 28734759 10.1016/S1470-2045(17)30422-9 PMC6207072

[58] Overman MJ , Lonardi S , Wong KYM , et al. Durable clinical benefit with nivolumab plus ipilimumab in DNA mismatch repair‐deficient/microsatellite instability‐high metastatic colorectal cancer. J Clin Oncol. 2018;36:773–9: JCO2017769901. 29355075 10.1200/JCO.2017.76.9901

[59] El‐Khoueiry AB , Sangro B , Yau T , et al. Nivolumab in patients with advanced hepatocellular carcinoma (CheckMate 040): an open‐label, non‐comparative, phase 1/2 dose escalation and expansion trial. Lancet. 2017;389:2492–502. 28434648 10.1016/S0140-6736(17)31046-2 PMC7539326

[60] Brahmer JR , Tykodi SS , Chow LQ , et al. Safety and activity of anti‐PD‐L1 antibody in patients with advanced cancer. N Engl J Med. 2012;366:2455–65. 22658128 10.1056/NEJMoa1200694 PMC3563263

[61] Lawrence MS , Stojanov P , Polak P , et al. Mutational heterogeneity in cancer and the search for new cancer‐associated genes. Nature. 2013;499:214–8. 23770567 10.1038/nature12213 PMC3919509

[62] Chalmers ZR , Connelly CF , Fabrizio D , et al. Analysis of 100,000 human cancer genomes reveals the landscape of tumor mutational burden. Genome Med. 2017;9:34. 28420421 10.1186/s13073-017-0424-2 PMC5395719

[63] Rizvi NA , Hellmann MD , Snyder A , et al. Cancer immunology. Mutational landscape determines sensitivity to PD‐1 blockade in non‐small cell lung cancer. Science. 2015;348:124–8. 25765070 10.1126/science.aaa1348 PMC4993154

[64] Bacher JW , Flanagan LA , Smalley RL , et al. Development of a fluorescent multiplex assay for detection of MSI‐High tumors. Dis Markers. 2004;20:237–50. 15528789 10.1155/2004/136734 PMC3839403

[65] Thibodeau SN , French AJ , Roche PC , et al. Altered expression of hMSH2 and hMLH1 in tumors with microsatellite instability and genetic alterations in mismatch repair genes. Cancer Res. 1996;56:4836–40. 8895729

[66] Hause RJ , Pritchard CC , Shendure J , Salipante SJ . Classification and characterization of microsatellite instability across 18 cancer types. Nat Med. 2016;22:1342–50. 27694933 10.1038/nm.4191

[67] Burkhart RA , Ronnekleiv‐Kelly SM , Pawlik TM . Personalized therapy in hepatocellular carcinoma: molecular markers of prognosis and therapeutic response. Surg Oncol. 2017;26:138–45. 28577719 10.1016/j.suronc.2017.01.009

[68] Prieto J , Melero I , Sangro B . Immunological landscape and immunotherapy of hepatocellular carcinoma. Nat Rev Gastroenterol Hepatol. 2015;12:681–700. 26484443 10.1038/nrgastro.2015.173

[69] Erkan M , Hausmann S , Michalski CW , et al. The role of stroma in pancreatic cancer: diagnostic and therapeutic implications. Nat Rev Gastroenterol Hepatol. 2012;9:454–67. 22710569 10.1038/nrgastro.2012.115

[70] Huang AC , Postow MA , Orlowski RJ , et al. T‐cell invigoration to tumour burden ratio associated with anti‐PD‐1 response. Nature. 2017;545:60–5. 28397821 10.1038/nature22079 PMC5554367

[71] Bazhin AV , Shevchenko I , Umansky V , Werner J , Karakhanova S . Two immune faces of pancreatic adenocarcinoma: possible implication for immunotherapy. Cancer Immunol Immunother. 2014;63:59–65. 24129765 10.1007/s00262-013-1485-8 PMC11028995

[72] Shirahama T , Muroya D , Matsueda S , et al. A randomized phase II trial of personalized peptide vaccine with low dose cyclophosphamide in biliary tract cancer. Cancer Sci. 2017;108:838–45. 28188670 10.1111/cas.13193 PMC5448649

[73] Kunisada Y , Eikawa S , Tomonobu N , et al. Attenuation of CD4(+)CD25(+) regulatory t cells in the tumor microenvironment by metformin, a type 2 diabetes drug. EBioMedicine. 2017;25:154–64. 29066174 10.1016/j.ebiom.2017.10.009 PMC5704053

[74] Walter S , Weinschenk T , Stenzl A , et al. Multipeptide immune response to cancer vaccine IMA901 after single‐dose cyclophosphamide associates with longer patient survival. Nat Med. 2012;18:1254–61. 22842478 10.1038/nm.2883

[75] Dauer M , Herten J , Bauer C , et al. Chemosensitization of pancreatic carcinoma cells to enhance T cell‐mediated cytotoxicity induced by tumor lysate‐pulsed dendritic cells. J Immunother. 2005;28:332–42. 16000951 10.1097/01.cji.0000164038.41104.f5

[76] Zheng Y , Xu M , Li X , Jia J , Fan K , Lai G . Cimetidine suppresses lung tumor growth in mice through proapoptosis of myeloid‐derived suppressor cells. Mol Immunol. 2013;54:74–83. 23220070 10.1016/j.molimm.2012.10.035

[77] Eikawa S , Nishida M , Mizukami S , Yamazaki C , Nakayama E , Udono H . Immune‐mediated antitumor effect by type 2 diabetes drug, metformin. Proc Natl Acad Sci USA. 2015;112:1809–14. 25624476 10.1073/pnas.1417636112 PMC4330733

[78] Mace TA , Shakya R , Pitarresi JR , et al. IL‐6 and PD‐L1 antibody blockade combination therapy reduces tumour progression in murine models of pancreatic cancer. Gut. 2018;67:320–32. 27797936 10.1136/gutjnl-2016-311585 PMC5406266

[79] Scheller J , Garbers C , Rose‐John S . Interleukin‐6: from basic biology to selective blockade of pro‐inflammatory activities. Semin Immunol. 2014;26:2–12. 24325804 10.1016/j.smim.2013.11.002

[80] He X , Guo X , Zhang H , Kong X , Yang F , Zheng C . Mechanism of action and efficacy of LY2109761, a TGF‐beta receptor inhibitor, targeting tumor microenvironment in liver cancer after TACE. Oncotarget. 2018;9:1130–42. 29416682 10.18632/oncotarget.23193 PMC5787425

[81] Lin TY , Lu CW , Wang CC , Huang SK , Wang SJ . Cyclooxygenase 2 inhibitor celecoxib inhibits glutamate release by attenuating the PGE2/EP2 pathway in rat cerebral cortex endings. J Pharmacol Exp Ther. 2014;351:134–45. 25047516 10.1124/jpet.114.217372

[82] Jiang H , Hegde S , Knolhoff BL , et al. Targeting focal adhesion kinase renders pancreatic cancers responsive to checkpoint immunotherapy. Nat Med. 2016;22:851–60. 27376576 10.1038/nm.4123 PMC4935930

[83] Fan Z , Xu X , Qi X , Wu Y . Role of TGF‐beta activated kinase‐1 inhibitor on the interaction between macrophages and mesangial cells on the condition of high glucose. Immunol Invest. 2018;47:303–14. 29373048 10.1080/08820139.2018.1428199

[84] Fujiki F , Oka Y , Tsuboi A , et al. Identification and characterization of a WT1 (Wilms Tumor Gene) protein‐derived HLA‐DRB1*0405‐restricted 16‐mer helper peptide that promotes the induction and activation of WT1‐specific cytotoxic T lymphocytes. J Immunother. 2007;30:282–93. 17414319 10.1097/01.cji.0000211337.91513.94

[85] Liu X , Shin N , Koblish HK , et al. Selective inhibition of IDO1 effectively regulates mediators of antitumor immunity. Blood. 2010;115:3520–30. 20197554 10.1182/blood-2009-09-246124

[86] Sharma P , Allison JP . The future of immune checkpoint therapy. Science. 2015;348:56–61. 25838373 10.1126/science.aaa8172

[87] Mehnert JM , Monjazeb AM , Beerthuijzen JMT , Collyar D , Rubinstein L , Harris LN . The challenge for development of valuable immuno‐oncology biomarkers. Clin Cancer Res. 2017;23:4970–9. 28864725 10.1158/1078-0432.CCR-16-3063 PMC5657536

[88] Ameratunga M , Chenard‐Poirier M , Moreno Candilejo I , et al. Neutrophil‐lymphocyte ratio kinetics in patients with advanced solid tumours on phase I trials of PD‐1/PD‐L1 inhibitors. Eur J Cancer. 2018;89:56–63. 29227818 10.1016/j.ejca.2017.11.012

[89] Galon J , Mlecnik B , Bindea G , et al. Towards the introduction of the ‘Immunoscore’ in the classification of malignant tumours. J Pathol. 2014;232:199–209. 24122236 10.1002/path.4287 PMC4255306

